# Clinician and Patient Perspectives on Cardiac Rehabilitation for Type 1 vs Type 2 Myocardial Infarction

**DOI:** 10.1016/j.jacadv.2026.103230

**Published:** 2026-09-22

**Authors:** Connor Suscha, Seán P. Murphy, Nhi Tong, Jeff C. Huffman, Elaine Luterstein, Avanthi Raghavan, Charlotte Paquette, Yuxi Liu, Jason H. Wasfy, Gheorghe Doros, Dalane W. Kitzman, Pradeep Natarajan, John A. Spertus, James L. Januzzi, Cian P. McCarthy

**Affiliations:** a Heart and Vascular Institute, Mass General Brigham, Boston, Massachusetts, USA; b Harvard Medical School, Boston, Massachusetts, USA; c School of Pharmacy, University of California, San Francisco, California, USA; d Department of Psychiatry, Massachusetts General Hospital, Boston, Massachusetts, USA; e Department of Medicine, Mass General Brigham, Boston, Massachusetts, USA; f Boston University School of Public Health, Boston, Massachusetts, USA; g Baim Institute for Clinical Research, Boston, Massachusetts, USA; h Wake Forest University School of Medicine, Winston-Salem, North Carolina; i Program in Medical and Population Genetics, Broad Institute of Harvard and MIT, Cambridge, Massachusetts, USA; j Center for Genomic Medicine, Massachusetts General Hospital, Boston, Massachusetts, USA; k University of Missouri-Kansas City’s Healthcare Institute for Innovations in Quality and Saint Luke's Mid America Heart Institute, Kansas City, Missouri, USA

**Keywords:** cardiac rehabilitation, type 1 myocardial infarction, type 2 myocardial infarction

## Abstract

**Background:**

Cardiac rehabilitation (CR) is recommended after myocardial infarction (MI), but perceptions of its role after type 2 MI remain unclear.

**Objectives:**

The aim of the study is to assess clinician and patient perceptions of CR benefits and safety after type 2 MI, clinician-reported referral frequency, and the willingness of patients to participate.

**Methods:**

In this cross-sectional study, 118 clinicians from 2,250 invitations across Mass General Brigham completed a survey comparing perceptions of CR after type 1 vs type 2 MI. Fifty type 2 MI inpatients at a single academic center completed a survey assessing perceptions of CR.

**Results:**

Among 110 clinicians with complete paired responses, fewer self-reported “always” or “often” referring patients after type 2 MI vs type 1 MI (30.0% vs 80.0%; paired difference: −50.0 percentage points; 95% CI: −59.4 to −40.2; *P* < 0.001), and fewer perceived CR as beneficial (69.1% vs 98.2%; paired difference: −29.1 percentage points; 95% CI: −38.2 to −21.4; *P* < 0.001). Most clinicians perceived CR as safe although slightly fewer rated it as safe after type 2 MI (92.7% vs 99.1%; paired difference: −6.4 percentage points; 95% CI: −12.6 to −2.8; *P* = 0.02). Among patients with type 2 MI, 90% perceived CR as beneficial, 80% believed CR would improve safety returning to activity, and 82% would likely attend if referred.

**Conclusions:**

Clinicians self-reported lower CR referral frequency after type 2 MI than after type 1 MI, with lower perceived benefit and barriers related to CR eligibility and referral process familiarity. Most patients with type 2 MI perceive CR as beneficial and report hypothetical willingness to participate if referred.

Approximately one million myocardial infarctions (MI) occur annually in the United States, with most cases classified as type 1 (atherothrombotic) or type 2 (supply-demand mismatch) MI.^1^ Both MI subtypes are associated with high rates of recurrent cardiovascular events,^2^, ^3^, ^4^ with higher all-cause mortality after type 2 MI, largely reflecting differences in comorbidity burden.^5^^,^^6^

Despite their cardiovascular risk, management of these MI subtypes differs substantially. Individuals with type 2 MI are less likely to be evaluated by a cardiologist and to receive therapies commonly used after type 1 MI, including antiplatelet therapy, lipid-lowering therapy, or other preventative medications.^6^, ^7^, ^8^, ^9^, ^10^ Persons with type 2 MI are also less likely to undergo anatomical or ischemic testing and revascularization.^2^ These differences highlight important gaps in evidence for this heterogeneous population and should not necessarily be interpreted as care deficiencies.

Given the uncertainty surrounding effective management approaches after type 2 MI, identifying interventions that may improve recovery and outcomes is critical. One potential approach is cardiac rehabilitation (CR), a comprehensive program designed to enhance post-MI recovery. CR includes exercise training, psychological counseling, and education; reduces cardiovascular events; and improves health status after MI.^11^^,^^12^

Despite these broad benefits among conventionally defined post-MI populations, the role of CR in patients with type 2 MI remains poorly defined. Existing studies suggest that individuals with type 2 MI are infrequently referred to CR.^13^^,^^14^ On one hand, CR may represent an important intervention for this population given the high prevalence of modifiable cardiovascular comorbidities and, on average, low levels of physical activity in this cohort.^2^^,^^3^ On the other hand, despite not being explicitly excluded, their representation in prior CR trials is uncertain.^11^ Limited but promising CR safety data exist specifically among individuals with type 2 MI.^14^

Understanding patients’ and clinicians’ perspectives may identify referral barriers and inform implementation strategies, while outcomes considered meaningful by both groups may guide future CR clinical trial design for type 2 MI.

We hypothesized that clinicians would be less likely to perceive CR as beneficial for patients after a type 2 MI than after a type 1 MI. The primary outcome was the proportion of clinicians who perceived CR as beneficial for patients with type 1 vs type 2 MI. Secondary clinician outcomes included self-reported referral frequency, perceived safety of CR, and reported barriers to CR referral. We also assessed patient-reported perceptions of CR benefit, hypothetical willingness to participate if referred, and perceived barriers to participation among individuals with type 2 MI.

## Methods

### Data sharing

The deidentified participant data, clinician and patient survey instruments (including the introductory definitions), and study protocol from this study have been submitted to the National Heart, Lung, and Blood Institute Biologic Specimen and Data Repository Information Coordinating Center (BioLINCC) repository (https://biolincc.nhlbi.nih.gov/). Researchers interested in accessing these materials should submit a data access request to BioLINCC, where requests will be reviewed in accordance with repository policies.

### Clinician survey

For this cross-sectional study, clinicians from 5 institutions within the Mass General Brigham hospital system—Massachusetts General Hospital (MGH), Brigham and Women’s Hospital (BWH), Newton Wellsley Hospital (NWH), Brigham and Women’s Faulkner Hospital, and Martha’s Vineyard Hospital (MVH)—were invited to participate. Potentially eligible clinicians (N = 2,250) included attendings, fellows, residents, and advanced practice providers, identified from hospital distribution lists for hospital/internal medicine (MGH, BWH, Brigham and Women’s Faulkner Hospital, NWH, and MVH), cardiology (MGH, BWH, NWH, and MVH), and cardiothoracic, thoracic, or vascular surgery (MGH and BWH). Potentially eligible clinicians were identified from departmental and divisional distribution lists provided by the participating services. Because the distribution listed was compiled independently and was not deduplicated, the number of unique eligible clinicians was unknown, and a response rate could not be calculated. Clinicians received an email invitation to complete an electronic survey if they self-reported previously providing care for a patient with MI.

The clinician survey was designed to assess how frequently clinicians refer patients with type 1 and type 2 MI to CR, as well as their perceptions of the benefit and safety of CR for each MI subtype and reasons for nonreferral. Clinicians were asked about their general referral practices, specifically the frequency with which they refer patients with type 1 or type 2 MI to CR at the time of hospital discharge or shortly thereafter. The survey did not further characterize the specific role of respondents in the referral process (eg, referral-order placement vs other aspects of referral coordination). The questions did not restrict responses to patients deemed clinically appropriate candidates for CR. Questions regarding type 1 and type 2 MI were presented in fixed order. An additional objective was to identify which outcomes from a potential clinical trial would most influence clinicians to refer patients with type 2 MI to CR. The clinician survey was developed by the study investigators based on the study objectives, including assessment of clinicians’ perceptions of CR benefit and safety, referral practices, barriers to referral, and outcomes considered important for future clinical trials. The survey was pilot tested among clinicians from various specialties and subspecialties to assess survey functionality, clarity, and content relevance. Ten clinicians completed pilot testing, and the survey was revised based on their feedback. The pilot data were not included in the final analysis. The finalized survey consisted of 17 questions related to study aims and 9 additional clinician characteristic questions.

The survey was distributed in March 2025, and responses were collected over an 8-week period. The clinician survey was hosted on REDCap (Research Electronic Data Capture) using secure institutional accounts that permitted anonymous participation. Two reminder emails were sent during the study period. To encourage participation, respondents could optionally provide contact information to enter a drawing for gift cards. Contact information was collected separately from survey responses and was not linked to response data. The drawing was conducted after the completion of the survey period.

### Patient survey

The cross-sectional patient survey was undertaken at MGH (Boston, Massachusetts). Over an 11-month period between March 2025 and February 2026, consecutively eligible inpatients with type 2 MI were identified by screening troponin data. Troponin measurements were obtained as part of routine clinical care using high-sensitivity cardiac troponin T (Roche Diagnostics) with a 99th percentile upper reference limit of 14 ng/L. Serial troponin measurements were obtained according to clinical practice. Acute myocardial injury was defined based on a rise or fall in troponin values, using a delta of 7 ng/L among patients with initially normal troponin values and a relative change of 20% among patients with elevated values.

On nonholiday weekdays, each troponin encounter was initially reviewed by one physician investigator (CMC, SM, AR, and EL) to adjudicate the encounter as type 1, 2, 4, or 5 MI, myocardial injury, or nonelevated troponin, using all available clinical, electrocardiographic, biomarker, and imaging data. Adjudication was performed according to the Fourth Universal Definition of the MI framework. Type 2 MI was distinguished from acute nonischemic myocardial injury by requiring evidence of myocardial ischemia in the setting of an acute supply-demand mismatch. Evidence of ischemia included ischemic symptoms, ischemic electrocardiographic changes, new regional wall motion abnormalities, or imaging evidence of new loss of viable myocardium. Cases with acute troponin elevation without evidence of ischemia were classified as acute myocardial injury rather than type 2 MI. Uncertain cases were reviewed by at least one additional physician investigator (C.M.C., J.J., J.W., or P.N.), and classification was determined through discussion until consensus was achieved. We excluded individuals who were: 1) younger than 18 years; 2) hemodynamically unstable; 3) pregnant or breastfeeding; 4) incarcerated; 5) were, or anticipated to be, discharged before being surveyed; 6) undergoing cancer treatment; 7) severely anemic (hemoglobin <8 g/dL); 8) had experienced an alternative MI subtype within the prior 6 months; 9) unable to provide informed consent; or 10) receiving palliative or comfort care only. Exclusion criteria were selected to identify patients for whom participation in CR after type 2 MI was expected to be feasible and clinically appropriate. Individuals undergoing active cancer treatment were excluded because competing treatment priorities and care needs could limit participation. Individuals with severe anemia were excluded because severe anemia may represent a condition requiring stabilization before consideration of CR activities. For eligible patients, the treating inpatient team was contacted and confirmed agreement with the likely diagnosis and granted permission to approach the patient. Patients were then invited to participate, and verbal informed consent was obtained. Consenting participants completed the one-time survey. The survey was administered by trained research coordinators (who were not involved in the adjudication process) through an interviewer-assisted format. Surveys were administered in English or with the assistance of a medical interpreter for participants who did not speak English. Interpreter services were available for both the informed consent process and survey administration. A total of 50 participants with type 2 MI were enrolled during the study period after weekday, nonholiday screening.

The patient survey was developed by the investigators to characterize the cardiovascular health of individuals with type 2 MI, assess their perceptions of the potential benefits and safety of CR, evaluate their likelihood of attending CR, determine preferred CR formats, and identify potential barriers to participation. The patient survey was developed by the investigator and not derived from previously validated instruments or formally validated through cognitive testing. Before answering questions regarding CR, participants were provided with the following description: “Cardiac rehabilitation is a personalized medically supervised program of exercise and education designed to improve your cardiovascular health. The program usually entails 1- to 3-hour-long sessions per week for 3 months involving exercise training, education about healthy living including diet, and stress reduction.” Participants were asked to select all CR formats they would be willing to attend. An additional question assessed which health outcomes were most meaningful to patients who experienced a type 2 MI. Participants were instructed to rank 6 health outcomes from most important (rank 1) to least important (rank 6), assigning a unique rank to each outcome. Ties were not permitted, and the order of response options was not randomized. The general cardiovascular health items, including questions regarding baseline physical activity, walking ability, physical or mental impairments, and fall history, were developed by the study investigators based on study objectives and intended to characterize participants’ self-reported functional status, activity level, and potential barriers to CR participation. These items were not derived from previously validated instruments and were not formally validated or subjected to cognitive testing. Participants were asked to report their typical weekly minutes of moderate- and vigorous-intensity physical activity prior to hospitalization. Moderate- and vigorous-intensity activities were defined using patient-facing descriptions based on the intensity of exertion and examples of common activities; responses were recorded as continuous minutes per week. The survey consisted of 11 core questions, 19 questions related to general cardiovascular health, and 16 supplementary questions related to demographic and social factors. The survey was hosted on REDCap. To encourage participation, respondents could opt into a gift card drawing conducted at the end of the survey period.

### Statistical analysis

The clinician survey sample size was based on the primary outcome comparing clinician perceptions of CR benefit for type 1 vs type 2 MI. Assuming that 90% of clinicians would perceive CR as beneficial for patients with type 1 MI, a sample of 200 clinicians caring for both MI subtypes would provide 80% power with a two-sided alpha of 0.05 to detect a 15% absolute difference in perceived benefit between type 1 and type 2 MI. During the study, we observed that a substantial proportion of respondents cared for both MI subtypes; therefore, the required number of clinicians contributing paired type 1/type 2 MI responses was lower than that initially anticipated. The patient survey was not designed to test a specific hypothesis; therefore, no formal sample size calculation was performed. The target sample size was selected to provide estimates of patient perceptions regarding CR benefit, willingness to participate, and potential barriers.

The primary clinician survey endpoint was the proportion of clinicians who perceived CR as beneficial for patients with type 1 vs type 2 MI. The primary analysis compared the proportion of clinicians selecting “very beneficial” or “beneficial” between MI subtypes among clinicians who reported caring for both type 1 and type 2 MI. Given that responses were paired within clinicians, comparisons were performed using McNemar’s test. Ordinal analyses of the full response distributions were considered secondary and performed using the Wilcoxon signed rank test. Secondary clinician outcomes included self-reported referral frequency and perceived safety of CR and reported barriers to referral. The patient survey was exploratory and descriptive. Analyses were performed to characterize patient perceptions of CR benefit, willingness to participate, preferred delivery formats, perceived barriers, and health outcomes considered most meaningful after type 2 MI.

Only complete survey responses were included in the analyses. Item-level missing data were not present among included clinician or patient survey respondents; therefore, no imputation methods were applied. Descriptive statistics were used to characterize the clinician and patient study participants. Categorical variables were summarized using counts and percentages, while continuous variables were summarized using mean ± SD or median (IQR).

For paired clinician analyses, we included clinicians who reported caring for both type 1 and type 2 MI and who provided complete paired responses for the CR referral, perceived benefit, and perceived safety questions. Ordinal responses were compared using the Wilcoxon signed rank test, and dichotomized responses were compared using McNemar’s test. Effect estimates for dichotomized outcomes were reported as paired differences in proportions with 95% CIs. Sensitivity analyses using chi-square tests were performed including all clinicians responding to each question. Dichotomized responses were “always/often” vs other responses, “very beneficial/beneficial” vs other responses, and “very likely/likely” vs other responses. Secondary clinician outcomes and exploratory patient survey analyses were not adjusted for multiple comparisons given the hypothesis-generating nature of these analyses. Results are presented with effect estimates and confidence intervals where applicable and should be interpreted accordingly. Analyses were conducted using R software, version 4.2.2 (R Foundation for Statistical Computing). All study procedures were approved by the Mass General Brigham Institutional Review Board (2024P003614).

## Results

### Clinician survey results

#### Clinician baseline characteristics

Invitations were distributed through departmental and divisional email distribution lists to approximately 2,250 potentially eligible clinicians. A total of 125 survey responses were received, corresponding to a crude completion proportion of 5.6% based on invitations distributed. Seven responses were incomplete and excluded in accordance with the prespecified analysis plan, leaving 118 complete surveys for analysis. Because invitations were not deduplicated across distribution lists and clinician eligibility among invitees was not verified, the number of unique eligible clinicians is unknown, and a true response rate could not be calculated. There was no item-level missingness among the included clinician surveys.

Among respondents, 63 (53.4%) were female, 85 were White (72.0%), and 30 (25.4%) were Asian. Just over half specialized in cardiovascular disease (66 [55.9%]), and approximately one-third specialized in internal medicine, hospital medicine, or primary care (37 [31.4%]). Most respondents were affiliated with MGH or BWH (104 [88.1%]). A diverse range of clinician roles and levels of experience were represented (Table 1). The vast majority of respondents treated both MI subtypes (111 [94.1%]). Clinicians estimated managing a median of 25 patients with type 1 MI annually (IQR: 5-78.75) and 50 patients with type 2 MI (IQR: 25-100).

**Table 1:** Characteristics of Clinician Survey Respondents (N = 118)

Demographics	
Sex	
Male, n (%)	55 (46.6)
Female, n (%)	63 (53.4)
Ethnicity	
Hispanic or Latino, n (%)	2 (1.7)
Race	
White, n (%)	85 (72.0)
Black or African American, n (%)	2 (1.7)
Asian, n (%)	30 (25.4)
Other, n (%)	1 (0.8)
Clinical characteristics	
Specialty	
Internal medicine, n (%)	110 (93.2)
Surgery or other specialty, n (%)	8 (6.8)
Subspecialty of internal medicine (if applicable)	
Cardiovascular disease, n (%)	66 (55.9)
Internal medicine/hospital medicine/primary care, n (%)	37 (31.4)
Other or not specified, n (%)	7 (5.9)
Subspecialty of surgery, if relevant n (%)	
Cardiothoracic surgery, n (%)	3 (2.5)
Vascular surgery, n (%)	1 (0.8)
Primary institution	
Massachusetts General Hospital, n (%)	66 (55.9)
Brigham and Women’s Hospital, n (%)	38 (32.2)
Brigham and Womens’ Faulkner Hospital, n (%)	3 (2.5)
Newton-Wellesley Hospital, n (%)	10 (8.5)
Other Mass General Brigham hospital, n (%)	1 (0.8)
Clinician role	
Attending, n (%)	65 (55.1)
Advanced practice provider, n (%)	30 (25.4)
Fellow, n (%)	15 (12.7)
Resident, n (%)	8 (6.8)
Years of practice	
<5 y, n (%)	18 (15.3)
5-10 y, n (%)	33 (28.0)
10-20 y, n (%)	32 (27.1)
21-30 y, n (%)	18 (15.3)
>30 y, n (%)	17 (14.4)
MI treated/managed	
Subtypes of MI managed/treated	
Type 1 MI only, n (%)	1 (0.8)
Type 2 MI only, n (%)	6 (5.1)
Both type 1 and type 2 MI, n (%)	111 (94.1)
Number of MIs managed per year	
Approximate type 1 MI patients managed per year, median (IQR)	25.00 (5.00, 78.75)
Approximate type 2 MI patients managed per year, median (IQR)	50.00 (25.00, 100.00)

MI = myocardial infarction.

#### Clinician perspectives regarding cardiac rehabilitation

Among the 111 clinicians who reported caring for both MI subtypes, 110 provided complete responses for all three paired clinician outcomes and were included in the paired analyses. In the paired analyses, clinicians self-reported less frequently referring patients with a type 2 MI to CR at the time of discharge, or during follow-up, compared with patients who experienced a type 1 MI (*P* < 0.001) (Central Illustration, Supplemental Table 1). Fewer reported “always” or “often” referring patients to CR after type 2 MI than after type 1 MI (33/110 [30.0%] vs 88/110 [80.0%]; paired difference: −50.0 percentage points, 95% CI: −59.4 to −40.2; *P* < 0.001). Clinicians perceived CR to be less beneficial for patients with type 2 MI than for those with type 1 MI (*P* < 0.001) (Central Illustration, Supplemental Table 2). Although most clinicians considered CR to be likely beneficial for type 2 MI, fewer perceived CR to be “very beneficial” or “beneficial” after type 2 MI compared with type 1 MI (76/110 [69.1%] vs 108/110 [98.2%]; paired difference: −29.1 percentage points; 95% CI: −38.2 to −21.4; *P* < 0.001). The overall response distribution favored a higher perceived safety of CR for type 1 MI than for type 2 MI (*P* < 0.001) (Central Illustration, Supplemental Table 3). Most clinicians perceived CR to be safe for both MI subtypes although the proportion rating CR as “very likely” or “likely” to be safe was slightly lower for type 2 MI (102/110 [92.7%] vs 109/110 [99.1%]; paired difference: −6.4 percentage points; 95% CI: −12.6 to −2.8; *P* = 0.02).

**Central Illustration:**
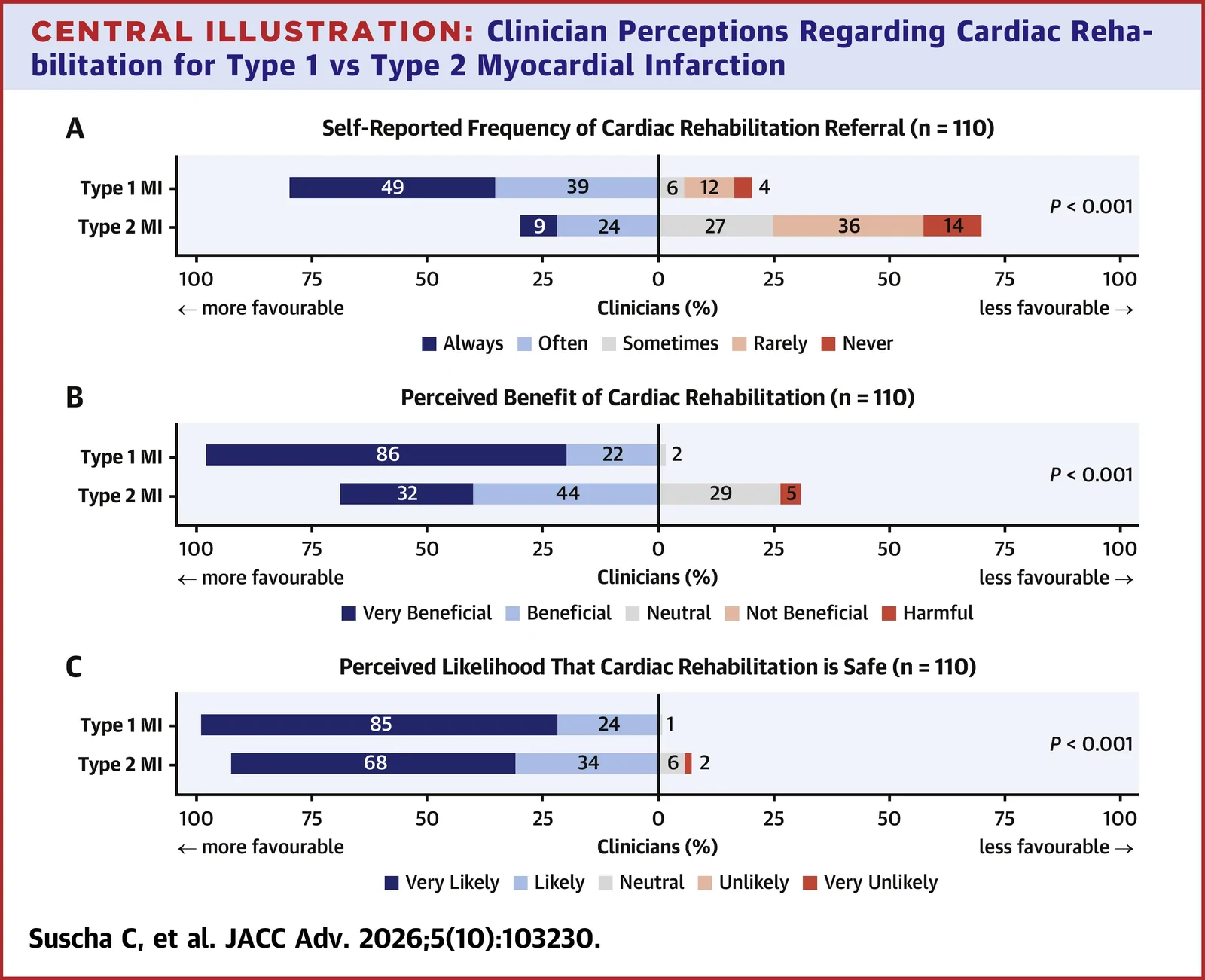
Clinician Perceptions Regarding Cardiac Rehabilitation for Type 1 vs Type 2 Myocardial Infarction Diverging Likert plots depicting paired clinician responses among clinicians caring for both MI subtypes, comparing perceptions of cardiac rehabilitation (CR) after type 1 vs type 2 myocardial infarction (MI): (A) frequency of clinician-reported CR referral at discharge or shortly after MI, (B) perceived benefit of CR, and (C) perceived safety of CR. Differences in paired responses between MI subtypes were assessed using the Wilcoxon signed rank test.

In a sensitivity analysis among all respondents (N = 118), clinicians reported referring patients with type 2 MI to CR less frequently than patients with type 1 MI (*P* < 0.001). Fewer clinicians self-reported “always” or “often” referring patients with type 2 MI to CR (33/117 [28.2%] vs 89/112 [79.5%]; *P* < 0.001). Clinicians also perceived CR as less beneficial for patients with type 2 MI (*P* < 0.001); fewer perceived it as “very beneficial” or “beneficial” (80/117 [68.4%] vs 110/112 [98.2%]; *P* < 0.001). Response distributions suggested higher perceived CR safety for type 1 MI than for type 2 MI (*P* = 0.04); however, this difference was not significant when responses were dichotomized as “very likely” or “likely” to be safe (109/117 [93.2%] vs 111/113 [98.2%]; *P* = 0.12).

Among clinicians who treat patients with type 2 or type 1 MI but self-reported not referring all eligible patients to CR (n = 89 and n = 111, respectively), the most commonly reported reasons for not referring are presented in Table 2. For type 1 MI, the more frequent barriers were patient preference (57.3%), absence of a local CR facility (40.4%), and lack of transportation (30.3%). By contrast, for type 2 MI, the most frequently reported reason was lack of awareness of eligibility for CR (35.1%), followed by patient preference (29.7%), and the presence of underlying comorbid conditions perceived to preclude participation (27.9%). Approximately 1 in five clinicians reported a lack of familiarity with the referral process.

**Table 2:** Reasons for Clinician Nonreferral to Cardiac Rehabilitation, Stratified by Myocardial Infarction Subtype

Type 1 MI (n = 89)	%	Type 2 MI (n = 111)	%
Patient preference	57.3	Lack of awareness of eligibility	35.1
Absence of local cardiac rehabilitation facility	40.4	Patient preference	29.7
Lack of transportation	30.3	Underlying condition precludes cardiac rehabilitation	27.9
Lack of familiarity with referral process	21.3	Insufficient evidence for cardiac rehabilitation	23.4
Lack of insurance/cost	21.3	Absence of local cardiac rehabilitation facility	21.6
Other	20.2	Lack of insurance/cost	19.8
Safety concern	6.7	Lack of familiarity with referral process	17.1
—	—	Other	14.4
—	—	Lack of transportation	10.8
—	—	Safety concern	4.5

Only clinicians who reported treating the specified myocardial infarction subtype and selected at least one reason for nonreferral (ie, a response other than “none—I refer all eligible patients”) were included. Respondents could select more than one reason for nonreferral; therefore, percentages do not sum to 100%. Denominators represent the number of clinicians providing responses for each myocardial infarction subtype.

When clinicians were asked to rank outcomes most likely to increase referral to CR for patients with type 2 MI if demonstrated in a clinical trial, reduction in all-cause mortality was most frequently ranked first, with 50.0% assigning it the top rank (Figure 1, Supplemental Table 4). An improvement in overall quality of life was the second most frequently highest ranked outcome. Greater variability in responses occurred for mid- and lower-tier ranks (Supplemental Table 4).

**Figure 1:**
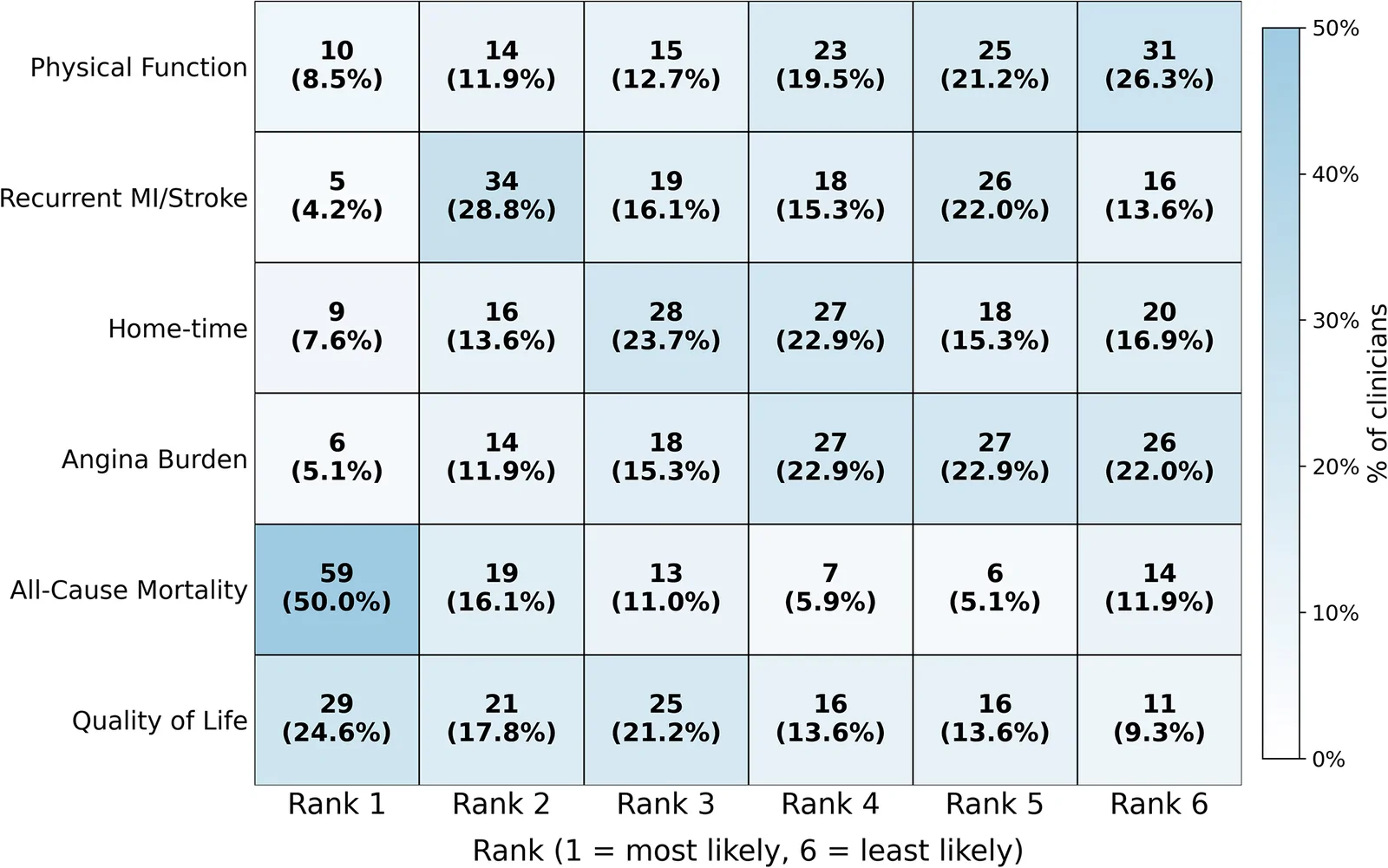
Heat Map of Clinical Trial Outcomes Influencing Clinician Referral to Cardiac Rehabilitation Heat map illustrating the forced-rank distribution of clinical trial outcomes that clinicians reported would most influence their likelihood of referring a patient with type 2 myocardial infarction to cardiac rehabilitation if improvement in the endpoint was demonstrated in a clinical trial. Participants assigned each outcome a unique rank from 1 (most likely to influence referral) to 6 (least likely to influence referral). Results are descriptive, and no inferential comparisons were performed. MI = myocardial infarction.

### Patient survey results

#### Patient baseline characteristics

Between March 16, 2025, and February 18, 2026, we identified 1,011 individuals with MI among whom 517 had type 2 MI. Among the 517 individuals with type 2 MI, 116 met eligibility criteria (Supplemental Figure 1). Of these, 50 were enrolled, while 66 were not enrolled. Reasons for nonenrollment included the treating team not granting permission to approach the patient (53 [80.3%]), patients’ refusal to participate (12 [18.2%]), and missed approach (1 [1.5%]). All 50 enrolled participants completed the survey, and there was no item-level missingness. Of the 50 participants, the average age was 68.8 years (standard deviation: 11.9 years), 24 (48.0%) were female, 38 (76.0%) were White, 7 (14.0%) were Black, 1 (2.0%) was Native Hawaiian or Pacific Islander, and 4 (8.0%) were Hispanic. Other participant characteristics are shown in Table 3.

**Table 3:** Characteristics of Patient Survey Respondents (N = 50)

Demographics	
Age, mean (SD)	68.8 (11.9)
Sex	
Male, n (%)	26 (52.0)
Female, n (%)	24 (48.0)
Ethnicity	
Hispanic or Latino, n (%)	4 (8.0)
Race	
White, n (%)	38 (76.0)
Black or African American, n (%)	7 (14.0)
Native Hawaiian or Pacific Islander, n (%)	1 (2.0)
Asian, n (%)	0 (0.0)
American Indian/Alaska Native, n (%)	0 (0.0)
Other, n (%)	4 (8.0)
Social factors	
Living alone, yes, n (%)	13 (26.0)
Highest level of education	
Less than a high school diploma, n (%)	8 (16.0)
High school diploma or GED, n (%)	7 (14.0)
Associate degree or technical school, n (%)	3 (6.0)
Some college but no degree, n (%)	3 (6.0)
Graduated from college with degree, n (%)	15 (30.0)
Postgraduate degree, n (%)	14 (28.0)
Prefer not to say or unknown, n (%)	0 (0.0)
Occupational status	
Paid full-time working, n (%)	9 (18.0)
Paid part-time working, n (%)	8 (16.0)
Not working for pay, n (%)	32 (64.0)
Prefer not to say or unknown, n (%)	1 (2.0)
Reasons not working for pay if applicable	
Retired, n (%)	25 (50.0)
Full-time homemaker, n (%)	2 (4.0)
Disabled, n (%)	1 (2.0)
Unpaid volunteer, n (%)	0 (0.0)
Unemployed but looking for work, n (%)	1 (2.0)
Unemployed and not looking for work, n (%)	3 (6.0)
Prefer not to say, n (%)	1 (2.0)
Type of health insurance^a^	
Private health insurance, n (%)	32 (64.0)
Medicare, n (%)	28 (56.0)
Medicaid/MassHealth, n (%)	15 (30.0)
VA health insurance, n (%)	0 (0.0)
Other, n (%)	1 (2.0)
Unknown or prefer not to say, n (%)	0 (0.0)
Overall difficulty getting medical care	
Extremely difficult, n (%)	0 (0.0)
Moderately difficult, n (%)	5 (10.0)
Somewhat difficult, n (%)	4 (8.0)
Not very difficult, n (%)	9 (18.0)
No problem at all, n (%)	32 (64.0)
If any difficulty getting care, major reasons	
Cost of care, n (%)	4 (8.0)
Difficulty finding a doctor, n (%)	7 (14.0)
Lack of insurance, n (%)	2 (4.0)
Travel and time, n (%)	8 (16.0)
Other, n (%)	6 (12.0)
Avoided health care services in past year because of cost, yes, n (%)	6 (12.0)
Economic burden of medical costs in past year	
Severe burden, n (%)	2 (4.0)
Moderate burden, n (%)	4 (8.0)
Somewhat of a burden, n (%)	5 (10.0)
A little burden, n (%)	0 (0.0)
Not a burden at all, n (%)	39 (78.0)
General cardiovascular health	
Level of activity before hospitalization	
Low (walking, light housework, or weeding), n (%)	28 (56.0)
Medium (walking briskly, cycling, or doing strenuous housework), n (%)	19 (38.0)
High (jogging/running, swimming, or doing heavy gardening), n (%)	3 (6.0)
Able to do a short (2-min) walk without use of a walking aid	
Yes, n (%)	39 (78.0)
No, n (%)	7 (14.0)
Unsure, n (%)	4 (8.0)
Physical/mental impairment precluding light to moderate exercise	
Yes, n (%)	9 (18.0)
No, n (%)	38 (76.0)
Unsure, n (%)	3 (6.0)
Number of falls in the past year	
None, n (%)	31 (62.0)
1-2 falls, n (%)	14 (28.0)
3-4 falls, n (%)	4 (8.0)
5 or more falls, n (%)	1 (2.0)
Minutes of moderate-intensity exercise per week, median (IQR)	70 (0-394)
Minutes of vigorous-intensity exercise per week, median (IQR)	0 (0-0)
Smoking status	
Current smoker, n (%)	3 (6.0)
Former smoker, quit within 1 year, n (%)	3 (6.0)
Former smoker, quit 1-5 y ago, n (%)	2 (4.0)
Former smoker, quit >5 y ago, n (%)	24 (48.0)
Never smoker, n (%)	18 (36.0)
Average hours of sleep per night, mean (STD)	6.5 (1.4)
Live independently, yes, n (%)	46 (92.0)
Overweight or obese (body mass index ≥25 kg/m^2^), n (%)	31 (62.0)
Adhere to specific diet^b^	
Mediterranean diet, n (%)	1 (2.0)
Vegetarian diet, n (%)	2 (4.0)
Ketogenic diet, n (%)	2 (4.0)
Intermittent fasting diet, n (%)	0 (0.0)
DASH or low salt diet, n (%)	12 (24.0)
Other, n (%)	3 (6.0)
No specific diet, n (%)	32 (64.0)
Self-reported history of high cholesterol, n (%)	27 (54.0)
Self-reported history of high blood pressure, yes, n (%)	37 (74.0)
Self-reported history of diabetes mellitus, yes, n (%)	10 (20.0)
Self-reported history of heart failure, yes, n (%)	16 (32.0)
Self-reported history of coronary artery disease, yes, n (%)	15 (30.0)
Self-reported history of heart attack, yes, n (%)	10 (20.0)
Self-reported history of kidney disease requiring dialysis, yes, n (%)	5 (10.0)

DASH = dietary approaches to stop hypertension; GED = General Educational Development; VA = Veterans Affairs.

a Participants could select more than one insurance type; therefore, percentages do not sum to 100%.

b Participants could select more than one diet type; therefore, percentages do not sum to 100%.

Just over half of the patients reported a low level of physical activity prior to hospitalization (28 [56.0%]), while 19 (38.0%) reported a medium level, and 3 (6.0%) reported a high level. Most patients self-reported the ability to undertake a short 2-minute walk without the use of a walking aid (39 [78.0%]), and a similar proportion stated they had no physical or mental impairments that might preclude them from light to moderate exercise (38 [76.0%]). Approximately two-thirds reported having no falls in the previous 12 months (31 [62.0%]). No participants had missing responses for moderate- or vigorous-intensity physical activity questions. Activity duration was reported as minutes per week and demonstrated a skewed distribution, with a median of 70 min/week (IQR: 0-394) for moderate-intensity activity and 0 min/week (IQR: 0-0) for vigorous-intensity activity.

On average, patients reported sleeping an average of 6.5 (standard deviation: 1.4) hours per night, and few were active smokers (3 [6.0%]). Nearly two-thirds were overweight or obese (31 [62.0%]). Most participants stated they followed no specific diet (32 [64.0%]), while almost one-quarter reported adhering to a low-salt diet (12 [24.0%]). Self-reported cardiovascular comorbidities included high blood pressure (37 [74.0%]), high cholesterol (27 [54.0%]), and diabetes mellitus (10 [20.0%]).

#### Patient perspectives regarding cardiac rehabilitation

Just over half of the participants reported no prior awareness of CR (27 [54.0%]). The vast majority stated they “agree” or “strongly agree” that CR would benefit their health (45 [90.0%]). Most “disagreed” or “strongly disagreed” that they have safety concerns about CR related to their cardiovascular disease (35 [70.0%]) or unrelated to their cardiovascular disease (39 [78.0%]). Approximately three-quarters “agreed” or “strongly agreed” that they are physically active enough to attend CR (37 [74.0%]), and 40 (80.0%) “agreed” or “strongly agreed” that participating in CR will make them feel safer about returning to physical activity. Most patients reported that if they were eligible for CR and were referred, they would “likely” or “very likely” attend (41 [82.0%]) (Table 4).

**Table 4:** Perspectives of Patients on Cardiac Rehabilitation (N = 50)

Number of participants who heard of cardiac rehabilitation previously, n (%)	23 (46.0)
Statement: “Cardiac rehabilitation will benefit my health”	
Strongly agree, n (%)	22 (44.0)
Agree, n (%)	23 (46.0)
Neutral/undecided, n (%)	3 (6.0)
Disagree, n (%)	2 (4.0)
Strongly disagree, n (%)	0 (0.0)
Statement: “I have safety concerns regarding cardiac rehabilitation because of my cardiovascular disease"	
Strongly agree, n (%)	1 (2.0)
Agree, n (%)	10 (20.0)
Neutral/undecided, n (%)	4 (8.0)
Disagree, n (%)	23 (46.0)
Strongly disagree, n (%)	12 (24.0)
Statement: ”I have safety concerns regarding cardiac rehabilitation unrelated to my cardiovascular disease“	
Strongly agree, n (%)	0 (0.0)
Agree, n (%)	7 (14.0)
Neutral/undecided, n (%)	4 (8.0)
Disagree, n (%)	26 (52.0)
Strongly disagree, n (%)	13 (26.0)
Statement: “I am active enough to attend cardiac rehabilitation”	
Strongly agree, n (%)	16 (32.0)
Agree, n (%)	21 (42.0)
Neutral/undecided, n (%)	6 (12.0)
Disagree, n (%)	4 (8.0)
Strongly disagree, n (%)	3 (6.0)
Statement: “Cardiac rehabilitation will make me feel safer/more confident about returning to physical activity”	
Strongly agree, n (%)	16 (32.0)
Agree, n (%)	24 (48.0)
Neutral/undecided, n (%)	7 (14.0)
Disagree, n (%)	3 (6.0)
Strongly disagree, n (%)	0 (0.0)
Statement: “If you were eligible for cardiac rehabilitation and your clinician referred you, how likely would you be to attend?”	
Very likely, n (%)	17 (34.0)
Likely, n (%)	24 (48.0)
Neutral, n (%)	5 (10.0)
Unlikely, n (%)	3 (6.0)
Very unlikely, n (%)	1 (2.0)
Statement: “If you had the opportunity to attend cardiac rehabilitation, in which format would you be willing to attend (select all)?”^a^	
At a local cardiac rehabilitation center (in person), n (%)	20 (40.0)
Home-based cardiac rehabilitation, n (%)	27 (54.0)
Hybrid, n (%)	25 (50.0)
Not applicable, would not attend, n (%)	4 (8.0)

a Participants could select more than one response option; therefore, percentages do not sum to 100%.

Across CR delivery formats, home-based CR was the most frequently selected format that participants reported they would be willing to attend (27 [54.0%]), followed by hybrid (25 [50.0%]) and in-person formats (20 [40.0%]) (Table 4). No participants selected “not applicable, I would not attend” in combination with another format option. The three most frequently listed barriers to attending CR were distance to a CR center (28 [56.0%]), financial concerns (18 [36.0%]), and time constraints unrelated to work (18 [36.0%]) (Supplemental Tables 5 and 6).

When patients were asked to rank a series of 6 health outcomes from most important to least important, improving one’s overall health-related quality of life was ranked first by 44.0% of respondents, with 72% ranking it in their top 2 priorities (Figure 2, Supplemental Table 7). The next most important outcomes to patients were avoiding a heart attack or stroke (20% rating it most important and 40% among the top 2 priorities) and survival (14% top, 42% as one of the top 2 prioritizes). Reducing or eliminating chest pain was the most least-important outcome to patients (21 [42.0%] rated it sixth). Other outcomes had variable ranks as shown in Supplemental Table 7. All enrolled participants completed the ranking task, with no incomplete or missing rankings.

**Figure 2:**
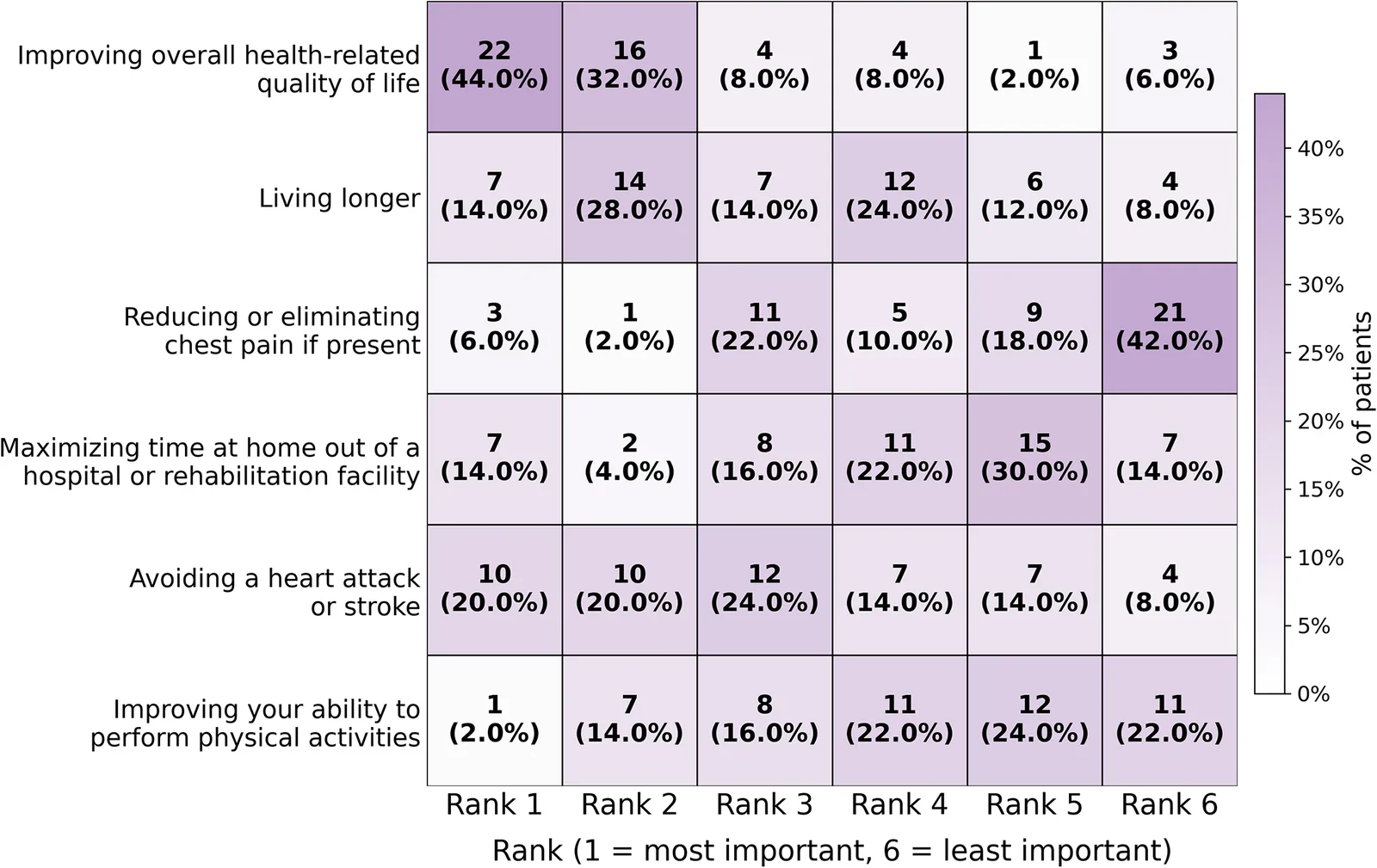
Heat Map of Health Outcomes Most Important to Patients With Type 2 MI Heat map illustrating the forced-rank distribution of health outcomes considered most important by patients with type 2 MI. Participants assigned each outcome a unique rank from 1 (most important) to 6 (least important). Results are descriptive, and no inferential comparisons were performed.

## Discussion

While prior research has demonstrated substantially lower use of CR for type 2 MI, to our knowledge, this is the first study to examine both clinician and patient perspectives on its use in this population. Clinicians across multiple hospitals and specialties reported substantially lower self-reported referral frequency for type 2 MI than for type 1 MI. Fewer than one-third of clinicians reported routinely referring patients with type 2 MI to CR despite most viewing CR as safe and potentially beneficial. These reported differences in referral frequency may reflect clinician-level barriers, including limited awareness of eligibility and referral processes. Separately, patients with type 2 MI in our survey expressed strong hypothetical willingness to attend CR if referred, providing complementary insight regarding their perspectives on CR participation.

Consistent with prior studies, clinicians in our cohort reported referring patients with type 2 MI to CR far less frequently than those with type 1 MI.^13^^,^^14^ Because clinical practice guidelines state that they are primarily applicable to type 1 MI,^15^^,^^16^ uncertainty remains regarding the extent to which recommendations, including those for CR, apply to individuals with type 2 MI. Clinicians’ responses provided insight into this management divergence. There was near-unanimous agreement regarding the benefits of CR for type 1 MI; however, only two-thirds of clinicians perceived CR to be beneficial for individuals with type 2 MI. As expected, there was a small gap between perceived benefit and referral for type 1 MI, likely reflecting case-specific barriers, with clinicians citing patient perspectives, limited local CR availability, and transportation as the most common obstacles to attendance. However, this gap was approximately twice as large for type 2 MI, suggesting that referral patterns in this population may additionally be influenced by factors beyond perceived effectiveness. Frequently cited barriers included uncertainty regarding patient eligibility, comorbidity burden, and unfamiliarity with the referral process. These findings suggest that some barriers to referral may be modifiable, in addition to highlighting the need for evidence on CR effectiveness in type 2 MI. Importantly, few clinicians reported concerns regarding CR safety in this population, although clinician perceptions do not establish the safety of CR after type 2 MI.

Patient responses reflected a more favorable perspective toward CR. Most patients with type 2 MI believed CR would benefit their health and expressed minimal concern about its safety. Furthermore, most agreed that CR would increase their confidence in returning to physical activity after hospitalization. Supporting this perceived benefit, modifiable cardiovascular risk factors such as obesity, hypertension, and diabetes were common, and most patients reported low levels of physical activity. Despite this, most respondents were able to engage in at least low levels of activity without a walking aid, underscoring the potential to improve functional capacity through CR.

Among patients, responses aligned closely with the goals of CR, with improvement in health-related quality of life identified as the most important outcome. By contrast, clinicians reported that evidence demonstrating a reduction in all-cause mortality would be more likely to influence their referral practices than improvements in outcomes such as quality of life. Together, these findings highlight differences in the outcomes prioritized by clinicians and patients in our study and underscore an opportunity for more deliberate goals-of-care discussions in clinical practice for this patient population. Similar differences between clinician- and patient-prioritized outcomes have been observed in cases of heart failure.^17^ These considerations may be particularly relevant after type 2 MI, a heterogeneous condition often occurring in the setting of substantial comorbidity burden and competing health priorities.

Despite the strong base of research supporting CR for the general post-MI population, the generalizability of previous CR trials to type 2 MI individuals remains unclear. Indeed, nearly one-quarter of clinicians reported a need for more evidence for CR for this population. Accordingly, further studies and trials examining CR implementation and effectiveness in type 2 MI would be welcome. Given the clinical heterogeneity of type 2 MI, future studies should also evaluate whether the feasibility, safety, and potential benefits of CR differ according to the mechanism of type 2 MI. One trial, the ATTEND-CR (Activating Teams and Patients to Enhance Attendance at Cardiac Rehabilitation After Secondary Myocardial Infarction; NCT07485491), will examine whether informational messaging to clinicians combined with motivational interviewing phone calls to patients increases CR attendance among individuals who experienced a type 2 MI. This implementation-focused pilot will provide preliminary data regarding feasibility, uptake, and the design of future studies evaluating the effectiveness and safety of CR in this population.

### Study Limitations

These results should be interpreted in the context of several limitations. First, generalizability is limited. Patient surveys were conducted at a single center, and detailed clinical characteristics were not collected for eligible patients who did not enroll; therefore, we could not assess whether enrolled participants differed systematically from nonenrolled patients or determine the extent to which findings represent the broader population of individuals with type 2 MI. In addition, enrolled patients had relatively high levels of educational attainment and reported limited financial barriers to health care access, which may affect perceptions of CR and willingness to participate. Therefore, patient perspectives observed in this cohort may not fully represent those of individuals with greater socioeconomic barriers or more limited access to care. Although clinician responses included multiple specialties and practice settings, perceptions may differ across institutions. Self-selection bias may have influenced both clinician and patient cohorts, as individuals with stronger opinions regarding CR may have been more likely to participate. Participation differed between clinician and patient surveys, which may also have influenced the representativeness of each cohort and should be considered when interpreting these findings.

Second, the survey instruments were developed for this study and were not formally validated. Although pilot tested and revised, the clinician survey was not formally assessed for validity. Patient responses may have been influenced by social desirability bias or limited CR familiarity despite a standardized program description.

Third, clinician survey responses reflect self-reported referral frequency rather than objective referral rates. The survey did not assess referrals among patients meeting specific CR eligibility criteria or distinguish among components of the referral pathway, such as referral-order placement, eligibility communication, or postdischarge coordination. Therefore, responses may reflect broader referral practices and clinical considerations regarding candidacy, comorbidity burden, and other factors influencing referral decisions. Anonymous survey links prevented the assessment of duplicate invitations or responses. We also did not collect information on email delivery, undeliverable invitations, or email opens and therefore could not assess potential nonresponse at these stages.

Finally, we did not collect the specific precipitating mechanisms of type 2 MI, limiting the assessment of whether perceptions of CR differed across clinical contexts or type 2 MI phenotypes. Although type 2 MI diagnoses were adjudicated using available clinical information and cases requiring additional review were resolved through consensus, we did not prospectively record the number of cases requiring a secondary review or assess interrater reliability.

## Conclusions

Among surveyed clinicians, self-reported referral frequency for CR after type 2 MI was substantially lower than the frequency for CR after type 1 MI and was accompanied by less certainty regarding its benefits and less familiarity with eligibility and referral processes. Among enrolled, clinically stable patients with type 2 MI, most expressed hypothetical willingness to participate in CR and confidence in its potential benefits after receiving a standardized description of the program. These findings underscore the need for implementation studies to better understand barriers to CR referral and participation, as well as clinical trials evaluating the effectiveness and safety of CR in individuals with type 2 MI.

Perspectives **COMPETENCY IN MEDICAL KNOWLEDGE:** Among surveyed clinicians, self-reported referral to cardiac rehabilitation was less frequent after type 2 myocardial infarction than after type 1 myocardial infarction. Lower self-reported referral frequency was accompanied by less certainty regarding the potential benefits of cardiac rehabilitation and less familiarity with eligibility and referral processes. Among enrolled, clinically stable patients with type 2 myocardial infarction, most expressed hypothetical willingness to participate after receiving a standardized description of cardiac rehabilitation.

**TRANSLATIONAL LOOK:** Future implementation studies should evaluate strategies to improve objective cardiac rehabilitation referral and participation after type 2 myocardial infarction and identify which type 2 myocardial infarction phenotypes are most appropriate for cardiac rehabilitation. These efforts should inform clinical trials evaluating the effectiveness and safety of cardiac rehabilitation using outcomes important to patients, including health-related quality of life and functional recovery.

## Funding support and author disclosures

This research was supported by the 10.13039/100000050National Heart, Lung, And Blood Institute under the award number K23HL167659 (PI; Dr McCarthy). The content is solely the responsibility of the authors and does not necessarily represent the official views of the National Institutes of Health. Dr Wasfy has been supported by the Massachusetts General Hospital Executive Committee on Research (24-1). Dr Januzzi is supported in part by the Adolph Hutter Professorship. Dr Natarajan reports research grants from Allelica, Amgen, Apple, Boston Scientific, Cleerly, Genentech/Roche, Ionis, Novartis, and Silence Therapeutics; personal fees from AIRNA, Allelica, Amgen, Apple, AstraZeneca, Bain Capital, Blackstone Life Sciences, Bristol Myers Squibb, Broadview Ventures, Creative Education Concepts, CRISPR Therapeutics, Eli Lilly & Co, Esperion Therapeutics, Foresite Capital, Foresite Labs, Genentech/Roche, GV, HeartFlow, Incyte, Magnet Biomedicine, Merck, Novartis, Novo Nordisk, TenSixteen Bio, Tourmaline Bio, and Ursa Medicines; equity in Bolt, Candela, Mercury, MyOme, Parameter Health, Preciseli, and TenSixteen Bio; royalties from Recora for intensive cardiac rehabilitation; and spousal employment at Vertex Pharmaceuticals, all unrelated to the present work. Dr Kitzman has received reimbursement as a consultant for and his institution has received reimbursement for clinical trials from Novo Nordisk, Bayer, and Rivus. He has served as a consultant for Lilly and Boehringer-Ingleheim and holds stock in Gilead. Dr Januzzi has received research grants from Abbott Diagnostics, Applied Therapeutics, AstraZeneca, HeartFlow, and Novartis; he has received consulting fees/honorarium from Abbott, AstraZeneca, Beckman-Coulter, Boehringer-Ingelheim, Bristol Myers, Intellia, Jana Care, Novartis, Pfizer, Merck, Roche Diagnostics, and Siemens; he participates on data safety monitoring boards or endpoint committees for Abbott, Abbvie, Bayer, CVRx, Pfizer, Roche Diagnostics, and Takeda; and reports equity holdings in Fibrosys, Imbria Pharmaceuticals, and Jana Care. Dr Spertus discloses providing consultative services on patient-reported outcomes and evidence evaluation to Janssen, Bristol Meyers Squibb, Terumo, Cytokinetics, BridgeBio, VentricHealth, and Imbria. He holds research grants from the National Institutes of Health, the Patient-Centered Outcomes Research Institute, the American College of Cardiology Foundation, Lexicon, Imbria, and Janssen. He owns the copyright to the Seattle Angina Questionnaire, Kansas City Cardiomyopathy Questionnaire, and Peripheral Artery Questionnaire and served on the Board of Directors for Blue Cross Blue Shield of Kansas City. Dr Wasfy has been supported by an interim deliberative support award from the Executive Committee on Research at the Massachusetts General Hospital. Dr McCarthy has received consulting fees or honorarium from Abbott Laboratories, Alnylam Pharmaceuticals, Azurity Pharmaceuticals, Inc, BioMerieux, Inc, Corcept Therapeutics, Cleerly Health, Heartflow, NewAmsterdam Pharma, and Roche Diagnostics. Dr Huffman receives salary from Elsevier for his role as the editor of General Hospital Psychiatry. All other authors have reported that they have no relationships relevant to the contents of this paper to disclose.
