# Supplementary material for: Clinician and Patient Perspectives on Cardiac Rehabilitation for Type 1 vs Type 2 Myocardial Infarction
Source: JACC Adv. 2026 Sep 22;5(10):103230. doi: 10.1016/j.jacadv.2026.103230 (PMC13634278; doi:10.1016/j.jacadv.2026.103230)
Supplement: Supplementary file 1 [file mmc1.docx]

**Supplemental Online Content**

Suscha C, Murphy SP, Tong N, et al. Clinician and Patient Perspectives on Cardiac Rehabilitation for Type 1 versus Type 2 Myocardial Infarction.

**Supplemental Table 1**: Paired responses for self-reported referral frequency by myocardial infarction subtype

|  | **Type 2 Myocardial Infarction** | | | | | | |
| --- | --- | --- | --- | --- | --- | --- | --- |
|  |  | **Always** | **Often** | **Sometimes** | **Rarely** | **Never** | **Total** |
| Type 1 Myocardial Infarction | **Always** | 8 | 13 | 13 | 12 | 3 | **49** |
|  | **Often** | 1 | 10 | 13 | 11 | 4 | **39** |
|  | **Sometimes** | 0 | 1 | 1 | 4 | 0 | **6** |
|  | **Rarely** | 0 | 0 | 0 | 9 | 3 | **12** |
|  | **Never** | 0 | 0 | 0 | 0 | 4 | **4** |
|  | **Total** | **9** | **24** | **27** | **36** | **14** | **110** |

**Supplemental Table 2**: Paired responses for perceived benefit by myocardial infarction subtype

|  | **Type 2 Myocardial Infarction** | | | | | |
| --- | --- | --- | --- | --- | --- | --- |
|  |  | **Very beneficial** | **Beneficial** | **Neutral** | **Not beneficial** | **Total** |
| Type 1 Myocardial Infarction | **Very beneficial** | 31 | 35 | 17 | 3 | **86** |
|  | **Beneficial** | 1 | 9 | 11 | 1 | **22** |
|  | **Neutral** | 0 | 0 | 1 | 1 | **2** |
|  | **Not beneficial** | 0 | 0 | 0 | 0 | **0** |
|  | **Total** | **32** | **44** | **29** | **5** | **110** |

**Supplemental Table 3**: Paired responses for perceived safety by myocardial infarction subtype

|  | **Type 2 Myocardial Infarction** | | | | | |
| --- | --- | --- | --- | --- | --- | --- |
|  |  | **Very likely** | **Likely** | **Neutral** | **Unlikely** | **Total** |
| **Type 1 Myocardial Infarction** | **Very likely** | 62 | 19 | 2 | 2 | **85** |
|  | **Likely** | 6 | 15 | 3 | 0 | **24** |
|  | **Neutral** | 0 | 0 | 1 | 0 | **1** |
|  | **Unlikely** | 0 | 0 | 0 | 0 | **0** |
|  | **Total** | **68** | **34** | **6** | **2** | **110** |

**Supplemental Table 4**: Ranking of clinical trial outcomes that clinicians reported would increase their likelihood of referring a type 2 MI patient to cardiac rehabilitation if an improvement in this endpoint was demonstrated in a clinical trial

| **Ranking** | **Improvement in overall quality of life (%)** | **Reduction in all-cause mortality (%)** | **Reduction in angina burden (%)** | **Increased days alive outside of the hospital or a facility (Home-time) (%)** | **Reduction in recurrent MI or non-fatal stroke (%)** | **Improvements in physical function (%)** |
| --- | --- | --- | --- | --- | --- | --- |
| 1 | 29 (24.6) | 59 (50.0) | 6 (5.1) | 9 (7.6) | 5 (4.2) | 10 (8.5) |
| 2 | 21 (17.8) | 19 (16.1) | 14 (11.9) | 16 (13.6) | 34 (28.8) | 14 (11.9) |
| 3 | 25 (21.2) | 13 (11.0) | 18 (15.3) | 28 (23.7) | 19 (16.1) | 15 (12.7) |
| 4 | 16 (13.6) | 7 (5.9) | 27 (22.9) | 27 (22.9) | 18 (15.3) | 23 (19.5) |
| 5 | 16 (13.6) | 6 (5.1) | 27 (22.9) | 18 (15.3) | 26 (22.0) | 25 (21.2) |
| 6 | 11 (9.3) | 14 (11.9) | 26 (22.0) | 20 (16.9) | 16 (13.6) | 31 (26.3) |

**Abbreviations**: MI= myocardial infarction

**Supplemental Table 5**: Factors listed by patients who experienced a type 2 MI as potential barriers to attending cardiac rehabilitation

| **Potential Barrier** | **N= 50** |
| --- | --- |
| Distance to cardiac rehabilitation center, n (%) | 28 (56.0) |
| Financial concerns, n (%) | 18 (36.0) |
| Time constraints unrelated to work, n (%) | 18 (36.0) |
| Lack of transportation, n (%) | 14 (28.0) |
| Physical impairment or injury, n (%) | 14 (28.0) |
| Family commitments, n (%) | 14 (28.0) |
| Lack of motivation, n (%) | 11 (22.0) |
| Time constraints related to work, n (%) | 9 (18.0) |
| None, n (%) | 5 (10.0) |
| Doubt about the benefit of cardiac rehabilitation, n (%) | 4 (8.0) |
| Language difficulties, n (%) | 4 (8.0) |
| Other, n (%) | 0 (0.0) |

**Supplemental Table 6**: Factors reported by patients who experienced a type 2 MI as the single biggest barrier to attending cardiac rehabilitation

| **Potential Barrier** | **N= 50** |
| --- | --- |
| Distance to cardiac rehabilitation center, n (%) | 10 (20.0) |
| Time constraints unrelated to work, n (%) | 7 (14.0) |
| Physical impairment or injury, n (%) | 7 (14.0) |
| Lack of transportation, n (%) | 5 (10.0) |
| None, n (%) | 5 (10.0) |
| Financial concerns, n (%) | 4 (8.0) |
| Time constraints related to work, n (%) | 3 (6.0) |
| Lack of motivation, n (%) | 3 (6.0) |
| Family commitments, n (%) | 3 (6.0) |
| Language difficulties, n (%) | 3 (6.0) |
| Doubt about the benefit of cardiac rehabilitation, n (%) | 0 (0.0) |
| Other, n (%) | 0 (0.0) |

**Supplemental Table 7:** Ranking of health outcomes most important to patients in terms of their health

| **Ranking** | **Improving your overall health related quality of life (%)** | **Living longer (%)** | **Reducing or eliminating chest pain if present (%)** | **Maximizing time at home out of a hospital or a facility such as a rehabilitation facility (%)** | **Avoiding a heart attack or stroke (%)** | **Improving your ability to perform physical activities (%)** |
| --- | --- | --- | --- | --- | --- | --- |
| 1 | 22 (44.0) | 7 (14.0) | 3 (6.0) | 7 (14.0) | 10 (20.0) | 1 (2.0) |
| 2 | 16 (32.0) | 14 (28.0) | 1 (2.0) | 2 (4.0) | 10 (20.0) | 7 (14.0) |
| 3 | 4 (8.0) | 7 (14.0) | 11 (22.0) | 8 (16.0) | 12 (24.0) | 8 (16.0) |
| 4 | 4 (8.0) | 12 (24.0) | 5 (10.0) | 11 (22.0) | 7 (14.0) | 11 (22.0) |
| 5 | 1 (2.0) | 6 (12.0) | 9 (18.0) | 15 (30.0) | 7 (14.0) | 12 (24.0) |
| 6 | 3 (6.0) | 4 (8.0) | 21 (42.0) | 7 (14.0) | 4 (8.0) | 11 (22.0) |

**Supplemental Figure 1**: Flow diagram illustrating eligibility and enrollment in the study
